# Dietary diversity and associated factors among children aged 6–23 months attending a public health hospital in Awi zone, Ethiopia, 2023

**DOI:** 10.3389/fnut.2024.1474995

**Published:** 2024-12-20

**Authors:** Sileshi Mulatu, Lemessa Jira Ejigu, Habtamu Dinku, Fikir Tadesse, Azeb Gedif, Fekiahmed Salah, Hailemariam Mekonnen Workie

**Affiliations:** ^1^Department of Pediatrics and Child Health Nursing, College of Medicine and Health Science, Bahir Dar University, Bahir Dar, Ethiopia; ^2^Department of Surgical Nursing, Collage Health Science, Pawie, Ethiopia; ^3^Department of Pediatrics and Child Health Nursing, Pawie Hospital, Pawie, Ethiopia; ^4^Department of Public Health, Pawie District Health Office, Pawie, Ethiopia

**Keywords:** children, dietary diversity, dietary practice, minimum dietary diversity, Ethiopia

## Abstract

**Background:**

Inadequate dietary diversity among children aged 6–23 months remains a public problem in Ethiopia. Adequate dietary diversity is crucial for children to meet their nutritional demands and promote healthy growth and development in infancy and young childhood.

**Objective:**

The study aimed to assess dietary diversity and associated factors among children aged 6–23 months in Awi Zone, Ethiopia, 2023.

**Methods:**

The study was conducted among children aged 6–23 months in Awi Zone, Amhara, Ethiopia, from August to September 2023. A community-based cross-sectional study design was conducted. A simple random sampling approach followed by face-to-face interview data collection techniques was used. To ascertain minimum dietary diversity, a 24 h food recall method comprising eight food item questionnaires was used. A statistical association was found between dependent and independent variables using the adjusted odds ratio with 95% confidence intervals and a *p*-value of ≤0.05.

**Result:**

This study found that only 192 (47.6%) children aged 6–23-month old had adequate dietary diversity. In this study, variables such as maternal education [AOR 2.36, 95% CI (1.297, 3.957)], birth interval [AOR 2.85, 95% CI (1.45, 4.25)], and food insecurity [AOR = 2.23, 95% CI (1.626, 3.1)] were strongly significant variables for the minimum dietary diversity of the child.

**Conclusion and recommendations:**

The proportion of the minimum dietary diversity was relatively low. Mother’s educational status, low birth intervals, and food insecurity were significant predictors of minimum dietary diversity. The stakeholders, including the Ministry of Health, regional health offices, and agricultural sectors, prioritize enhancing child nutrition through targeted food-based approaches. Developing and implementing comprehensive intervention programs to improve children’s minimum dietary diversity (MDD) should be a central focus. Professionals should strengthen nutrition education to promote optimal MDD practices.

## Introduction

Dietary diversity refers to a variety of food types that are frequently used to gauge the variety and nutrient sufficiency of diets (1). Increasing the variety of foods and food groups for a child can ensure the adequacy of essential nutrients (2, 3). Food categories from various diets are crucial for child-feeding techniques that meet their nutritional demands and promote healthy growth and development in infancy (4, 5). For a child to experience the best possible growth and development, proper feeding methods for infants and young children are essential (6). In addition, a lack of diversified foods puts children at a greater risk of not achieving their potential growth and development, dropping out of school, and failing classes, which has a significant negative impact on communities, families, and educational institutions (7, 8).

The first 2 years of a child’s life are crucial for their growth and development, making proper infant and young child-feeding (IYCF) practices essential (9–11). Inappropriate feeding during this period is linked to over half of the under-five child mortality cases (12). Adequate nutrition in these early years is vital not only for physical growth but also for mental development and long-term health (13).

To improve IYCF practice and address these critical needs, the WHO-UNICEF Technical Expert Advisory Group on Nutrition Monitoring (TEAM) has recommended revising the minimum dietary diversity (MDD) indicator (14, 15). They have developed a set of core indicators to better assess feeding practices for children aged 6–23 months, emphasizing both breastfeeding and complementary feeding. These indicators aim to ensure that children receive the necessary nutrients to reduce morbidity, mortality, and the risk of chronic diseases (16–19).

Minimum dietary diversity (MDD) measures the variety of foods or food groups ingested during a 24 h period (17). The WHO-UNICEF Technical Expert Advisory Group identified eight food groups that provide the necessary amount of macro- and micro-nutrients for children aged 6–23 months: breast milk, grains, roots, and tubers; legumes and nuts; dairy products; flesh foods (meats, fish, and poultry); eggs; vitamin A-rich fruits and vegetables; and other fruits and vegetables. Children aged 6 to 23 months are advised to eat at least five different food groups every day (4, 20).

Globally, only 28.2% of children aged 6–23 months achieve the recommended level of dietary diversity (21). This issue is exacerbated in low- and middle-income countries (LMICs), particularly in regions such as South Asia and parts of Africa (22). Despite efforts to improve dietary diversity, Ethiopia still reports the lowest level of adequate dietary diversity among East African countries (23, 24). In addition, stunting, underweight, and chronic diseases in children are closely linked to adequate dietary diversity (25). Children who are stunted, underweight, or with chronic diseases are less likely to meet dietary diversity requirements and are more susceptible to infections and illnesses (26). Evidence-based nutritional information, by providing scientifically proven guidelines and recommendations specifically targeted to the needs of various age groups, plays a critical role in enhancing infant and young child-feeding (IYCF) practices and reducing childhood malnutrition (27, 28). With this knowledge, common nutritional deficiencies can be correctly identified and addressed, feeding techniques can be suggested, and a varied, nutrient-rich diet can be encouraged (29, 30). It guarantees the knowledge of healthcare providers and caregivers, helps in the monitoring and assessment of nutrition treatments, and supports the creation of focused public health policies and programs (31). Evidence-based knowledge improves the efficacy of initiatives to address under-nutrition, over-nutrition, and associated health problems by firmly establishing nutrition recommendations in scientific research, eventually improving children’s health outcomes (32). The Ethiopian government has launched a multifaceted strategy to boost dietary diversity and improve child nutrition, centered on the National Nutrition Program (NNP), which promotes diverse food consumption and behavior change communication (33).

This study aimed to determine the prevalence of dietary diversity feeding practices and their determinants among children aged 6–23 months in the Awi Zone. The findings will inform nutritional education and counseling for mothers and caregivers about the importance of IYCF practice. In addition, this finding will provide insights for governmental and non-governmental intervention to address under-nutrition and mitigate its long-term impact.

## Methods

### Study design, area, and period

The institution-based cross-sectional study was conducted in Awi Zone, Amhara Regional State, from two public hospitals from 1 August to 28 September 2023.

Awi Zone is one of the 11 zones in the Amhara Region. This zone includes three town administrations and nine rural woredas.

It is 439.4 km away from Addis Ababa, the capital city, and 114 km away from Bahir Dar city, the capital city of the region, which is a well-productive and comfortable zone for agriculture. According to the information from the Zone Health Office, there are three public hospitals and many governmental and private health centers that provide health services for the community. The study was carried out in two selected public hospitals in the Awi Zone (Injibara and Dangila Hospitals), which serve the majority of the population in the Awi Zone.

### Source population

Children aged 6–23 months paired with mothers/caregivers in the Awi Zone.

### Study population

Children aged 6–23 months paired with mothers/caregivers who received health services in the selected hospital.

### Inclusion and exclusion criteria

#### Inclusion criteria

Children aged 6–23 months paired with mothers/caregivers who received health services during the data collection period in the selected study area.

#### Exclusion criteria

Children aged 6–23 months paired with mothers/caregivers who were critically ill and unable to feed or children who were on tube feeding in the last 24 h.

### Sample size and sampling technique

The sample size was calculated by using the single population proportion formula. The proportion of dietary diversity practice was estimated to be 59.9% in a study conducted in Addis Ababa (34). Therefore, the sample size was determined using the following assumptions: proportion (p) 59.9%, margin of error (d) 5%, and Zα/21.96 with 95% CI.


$$N=\frac{{\left(Z\alpha /2\right)}^{2}\times P\;\left(1-P\right)}{d^{2}}=\frac{{\left(1.96\right)}^{2}\times 0.599\left(1–0.599\right)}{{\left(0.05\right)}^{2}}$$

n=369

By adding 10% non-response, which is 37, the final sample size was **406.**

### Sampling technique and procedures

To contact study participants, a multistage sampling methodology that combined simple random and systematic random sampling methods was used. The sampling frame for the initial phase included all hospitals in the Awi Zone. A lottery method was used to choose Enjbara and Dangila hospitals. After that, the proportional allocation was used to divide the 406 people in the sample size evenly across the two hospitals that were chosen. All the study participants received comprehensive information regarding the goals and methods of the study. All participants gave their informed consent prior to involvement, confirming that they were aware of their rights, that their participation was voluntary, and that they may leave at any moment without incurring any fees; this procedure followed ethical guidelines.

### Data collection procedures

Data were collected from mothers or caregivers who had children aged 6–23 months from each household by direct interviewing. Face-to-face interviews were conducted by a trained team using a structured guide and standardized procedures, with rigorous monitoring and supervision to ensure consistency and reliability. A per-tested, interviewer-administered questionnaire was used to collect data from child mothers/caregivers who had children aged 6–23 months of age. For this study, a validated dietary assessment tool was used to ensure the accurate and reliable measurement of dietary diversity. The tool was developed by the WHO-UNICEF Technical Expert Advisory Group on Nutrition Monitoring (TEAM) (19). Minimum dietary diversity/recommended dietary diversity was defined as consuming five or more food types or groups in the preceding day/24 h/ of the survey out of the eight standard food groups that were recommended by the WHO (35). The questionnaire was prepared in English, and for fieldwork purposes, the questionnaires were translated into the Amharic version and then translated back into the English version. The questionnaire contains four parts: socio-demographic characteristics, child and maternal health services utilization, household food security, and dietary diversity assessment tools. The MDDs were assessed by asking the child’s mother/caregiver whether the child consumed the WHO-recommended food group on the previous day of the survey. The data were collected by eight trained nurses and supervised by two supervisors.

### Data quality control

The questionnaire was prepared in English and translated to the local language (Amharic version). Interviewers were trained on the aim of the research, the content of the questionnaire, and how to conduct interviews to increase their performance in field activities before data collection, and a pretest was conducted on 5% of child mothers/caregivers before actual data collection outside the selected rural kebeles. Two-day training was given for the data collectors, and the collected data were checked every day by the principal investigator.

### Data processing and analysis

After the completion of the data, the data were cleaned, coded, and entered into EpiData version 7, and then transformed to SPSS version 26 for analysis. Data analysis was performed using SPSS statistical software where adjusted odds ratios and confidence intervals were calculated, and additional tests such as chi-square tests were conducted to assess the associations and ensure the robustness of the findings.

Multi-collinearity was assessed to check whether independent variables in a regression model are highly correlated with each other, and an effort was made to incorporate different models to cross-check.

The results of the study are presented in text, tables, and graphs. Frequency and cross-tabulation were calculated to describe the study population about relevant variables. A binary logistic regression was performed to select the variables for a multivariate analysis. A multi-variable logistic regression analysis was performed on the variables with a *p*-value <0.25. Before adjusting in the multi-variable analysis, the variable candidates for the multi-variable analysis were checked for multi-collinearity using the variance inflation factor, and the VIF was less than 10, which was acceptable. A multi-variable logistic regression analysis was performed to identify the independent predictors of the minimum dietary diversity. The Hosmer–Lemeshow test was used to assess the model’s fitness [0.124]. *p*-values <0.05 were considered statistically significant, and an adjusted odds ratio (AOR) with a 95% confidence interval was used to measure the degree of association.

### Operational definitions

#### Adequate dietary diversity

Proportion of children 6–23 months of age who receive five food groups from eight food groups during the previous days of data collection. The eight food groups used for tabulation of this indicator were grains, roots, and tubers; legumes and nuts; dairy products (milk, yogurt, and cheese); flesh foods (meat, fish, poultry, and liver/organ meats); eggs; vitamin A-rich fruits and vegetables; and other fruits and vegetables. Children who consumed less than five food groups were considered to have inadequate dietary diversity.

#### Inadequate dietary diversity

Proportion of children 6–23 months of age who receive four and less than four food groups from eight food groups during the previous days of data collection.

### Variables

#### Dependent variables

Child’s dietary diversity.

#### Independent variables

Socio-demographic characteristics: child age, child sex, mother’s education, father’s education, mother’s occupation, father’s occupation, mother’s age, birth order, marital status, religion, income/wealth, food security, and parity.

Child characteristics: gender, birth order, gestational age at birth, feeding problem, duration of Breast Feeding, frequency of feeding, and duration of exclusive BF. Health-related factors: starting date of complementary feeding, birth interval, antenatal care, postnatal care, education on how to feed children, place of delivery, and education on how to feed children.

### Ethical consideration

Ethical approval was obtained from the Ethical Review Board of Bahir Dar University College of Medicine and Health Science. Then, a permission letter was acquired from the College of Health Research Management Directorate for the Awi Zone Health Office, and the office wrote the letter for the selected public hospital. Written informed consent was obtained from the study participants. The participant’s strict confidentiality was ensured and their identity was not shown; there was no dissemination of the information without the respondent’s permission. The data given by the participants were used only for research purposes.

## Results

### Socio-demographic characteristics of mothers and children

A total of 403 mothers with children aged 6–23 months were interviewed, achieving a response rate of 99.26% because recruitment efforts involved clear communication about the study’s purpose, ensuring informed consent, and adhering to rigorous standards to avoid conflicts of interest. Among the participants, 38.5% had children aged 13–18 months, and more than half (53.6%) of the children were male individuals. Regarding religious affiliation, 212 participants (52.6%) were Orthodox.

Educationally, 115 mothers (28.5%) had attained a certificate or higher, while 181 participants (44.9%) were housewives. In addition, 175 participants (43.4%) had families with 4–5 members, and 185 participants (45.9%) reported a monthly income greater than 3,001 ETB (Table 1).

**Table 1:** Socio-demographic characteristics of children aged 6–23 months in Awi Zone, Ethiopia, 2023 (*N* = 403).

Background characteristics	Frequencies	Percentage (%)
Age of the child in months		
6–12 months	105	26.1
13–18 months	155	38.5
19–23 months	143	35.5
Sex of the child		
Male	216	53.6
Female	187	46.4
Age of the mother (in years)		
<20 years	48	11.9
20–29 years	186	46.2
30–39 years	149	37.0
40 and above	20	5.0
Ethnicity (*n* = 403)		
Amhara	286	70.9
Agew	117	29.1
Religion (*n* = 403)		
Orthodox	212	52.6
Muslim	152	37.7
Protestant	27	6.7
Catholic	12	3.0
Marital status (*n* = 403)		
Married	364	90.3
Single	11	2.7
Divorced	28	6.9
Maternal educational level (*n* = 403)		
Unable to read and write	76	18.9
Primary	92	22.8
Secondary	120	29.8
Certificate and above	115	28.5
The educational level of the father		
Unable to read and write	69	17.1
Primary	77	19.1
Secondary	117	29.0
Certificate and above	140	34.7
Maternal occupation status (*n* = 403)		
Housewife	181	44.9
Government employee	118	29.3
Merchants	89	22.1
Others	15	3.7
Occupation status of the father (*n* = 403)		
Government employee	145	36.0
Merchant	125	31.0
Self-employed	97	24.1
Others	36	8.9
Income (in ETB) (*n* = 403)		
<1,000 ETB	33	8.2
1,000–2000 ETB	81	20.1
2001–3,000 ETB	104	25.8
>3,001 ETB	185	45.9
Family size (*n* = 403)		
2–3	113	28.0
4–5	175	43.4
6 and above	115	28.5

### Maternal and child health characteristics

Of the total study participants, 121 (30%) had a birth interval greater than 24 months, and 96 (23.6%) lived in households with two children under 5 years. *Of* the total, 363 (90.1%) of children were fully immunized, and the majority (82.4%) of the children were full-term babies. Nearly two-thirds (61.5%) of the mothers had fourth visit antenatal care (ANC) follow-up, and almost all (94.3%) of mothers gave birth at the health facility. Three hundred (74.4%) mothers had postnatal care (PNC) follow-up. A small number (6.5%) of the children had a feeding problem, and only 49 (12.2%) children had a history of illness in the previous 2 weeks. The majority (86.4%) of the children started receiving complementary feeding after the age of 6 months (Table 2).

**Table 2:** Maternal and child health characteristics of children aged 6–23 months in Awi Zone, Ethiopia, 2023 (*N* = 403).

Maternal and child health characteristics	Number	Percent (%)
Birth interval		
No previous birth	150	37.2
<24 Months	132	32.8
>24 Months	121	30.0
Number of < 5		
One	307	76.2
Two	96	23.8
Immunization of the child		
Yes	363	90.1
No	40	9.9
Gestational age of the child		
<32 weeks	13	3.2
32–36 weeks	32	7.9
37–42 weeks	332	82.4
>42 weeks	26	6.5
ANC follow-up in the last pregnancy		
None	23	5.7
1 visit	15	3.7
2–3 visits	117	29.0
4 and above	248	61.5
Place of delivery		
Hospital	257	63.8
Home	23	5.7
Health center	123	30.5
PNC service		
No	103	25.6
Yes	300	74.4
Does your child have any feeding problem?		
No	377	93.5
Yes	26	6.5
Does your child have a history of illness in the previous 2 weeks?		
No	354	87.8
Yes	49	12.2
Starting date of complementary feeding		
After 6 months	348	86.4
Before 6 months	55	13.6

### Dietary diversity of the 6–23 months of children

Of the total, 324 (80.4%) mothers breastfed their children. In addition, more than three-fourths of the mothers, 314 (77.9%), reported consuming dairy products (milk, yogurt, and cheese) in the previous 24 h. More than two-thirds of the children consumed grains, roots, and tubers, as well as eggs, within the same time frame. Similarly, two-thirds of the children consumed flesh foods (meat, fish, poultry, liver, or other organs), while only one-third consumed legumes and nuts. Over three-quarters and two-thirds of the children did not consume vitamin A-rich foods (fruits and vegetables) and other fruits and vegetables, respectively, in the previous 24 h. Based on these categories, 197 (42.6%) children exhibited adequate dietary diversity (DD), defined as consuming five or more food groups, while the remaining 52.4% fell into the low DD category, consuming fewer than five food groups (Table 3).

**Table 3:** MDDs among children aged 6–23 months in Awi Zone, Ethiopia, 2023 (*N* = 403).

Food group consumption in the previous 24 h	Number	Percent
Breastfeeding		
No	79	19.6
Yes	324	80.4
Dairy products (milk, yogurt, and cheese) in the previous 24 h.		
No	89	22.1
Yes	314	77.9
Grains, roots, and tubers in the previous 24 h.		
No	135	33.5
Yes	268	66.5
Eggs in the previous 24 h.		
No	141	35.0
Yes	262	65.0
Flesh foods (meat, fish, poultry, liver, or other organs) in the previous 24 h.		
No	158	39.2
Yes	245	60.8
Legumes and nuts in the previous 24 h.		
No	259	64.3
Yes	144	35.7
Vitamin A-rich fruits and vegetables in the previous 24 h.		
No	309	76.7
Yes	94	23.3
Other fruits and vegetables in the previous 24 h.		
No	257	63.8
Yes	146	36.2
Child’s dietary diversity score		
Inadequate child dietary diversity	211	52.4
Adequate child dietary diversity	192	47.6

### Food security characteristics of the HH

As shown in Table 4, the food security status of households emerged as a crucial factor influencing the dietary diversity of children aged 6–23 months. Among the nine Household Food Insecurity Access Scale (HFIAS) items, a significant majority of households, 331 (82.1%), expressed concerns about running out of food. In addition, 322 (79.9%) households reported being unable to consume their preferred foods. Furthermore, 343 (85.1%) households were found to have a limited variety of food choices. More than half of the households resorted to eating food they did not prefer, and over a quarter skipped meals within the past 24 h. The overall prevalence of food insecurity was notable, with 334 (41.4%) households classified as food insecure (Table 4).

**Table 4:** HFIAS items of children aged 6–23 months in Awi Zone, Ethiopia, 2023 (*N* = 403).

Characteristics	Number	Percent
Worried about running out of food		
Yes	331	82.1
No	72	17.9
Unable to eat preferred foods		
Yes	322	79.9
No	81	20.1
Eat a limited variety of foods		
Yes	60	14.9
No	343	85.1
Eat foods that you did not want to eat		
Yes	355	88.1
No	48	11.9
Eat a smaller meal		
Yes	214	53.1
No	189	46.9
Skipping meals		
Yes	108	26.8
No	295	73.2
No food to eat of any kind in the household		
Yes	17	4.2
No	386	95.8
Go to sleep at night hungry		
Yes	22	5.5
No	381	94.5
Go a whole day and night without eating anything		
Yes	14	3.5
No	389	96.5
Food security status		
Food secure	249	61.8
Food insecure	154	38.2

### Factors associated with MDDs of children aged 6–23 months

Table 5 shows bi-variable and multi-variable analysis of factors associated with MDDs of a child aged 6–23 months. In the binary logistic regression, only ten variables had a *p*-value less than 0.25, and multi-variable logistic regression was run for all these ten variables. In the multi-variable logistic analysis, only three variables (maternal education, birth interval, and food security) had scientifically significant factors for the outcome variables. In terms of maternal education status, mothers with a secondary education and above were 2.36 times [AOR 2.36, 95% CI (1.30, 3.96)] more likely to have adequate dietary diversity than those who were unable to read and write. On the other hand, birth interval was one significant variable that affected the minimum dietary diversity of the child. Mothers with birth intervals greater than 24 months were 2.85 times [AOR 2.85, 95% CI (1.45, 4.25)] more likely to practice adequate dietary diversity than those with birth intervals less than 24 months. Finally, food insecurity is also one scientifically significant factor that affects the minimum dietary diversity of the children. Food-secure households were 2.23 times more likely to practice adequate dietary diversity than households with food insecurity [AOR = 2.23, 95% CI (1.63, 3.10)] times more likely to feed diversified food to their children than their counterparts (Table 5).

**Table 5:** Factors associated with MDD among children (*n* = 403) at Awi Zone, Ethiopia, 2023.

Variables	DDs		COR at 95%CI	AOR at 95%CI
	Poor	Good		
Age of the child				
6–12 months	56	49	0.938 (0.57, 1.55)	0.520 (0.22, 1.20)
13–18 months	81	74	0.980 (0.62, 1.54)	0.630 (0.27, 1.46)
19–23 months	74	69	1	1
Age of the mother (in years)				
<20 years	21	27	0.857 (0.30, 2.48)	1.740 (0.69, 4.40)
20–29 years	108	78	0.481 (0.19, 1.23)	0.880 (0.38, 2.05)
30–39 years	74	75	0.676 (0.26, 1.75)	0.900 (0.39, 2.05)
40 and above	8	12	1	1
Maternal educational level				
Unable to read and write	40	36	**1**	**1**
Primary	38	54	1.210 (0.68, 2.17)	1.220 (0.47, 3.13)
Secondary	67	53	**1.910 (1.10, 3.34)**	**2.360 (1.30, 3.96)**
Certificate and above	66	49	**2.070 (1.64, 2.78)**	**2.590 (1.26, 3.32)**
Educational level of the father				
Unable to read and write	34	35	1.130 (0.73, 1.74)	0.520 (0.22, 1.20)
Primary	35	42	1.258 (0.71, 2.24)	0.630 (0.27, 1.46)
Secondary	65	52	1.467 (0.84, 2.56)	0.510 (0.23, 1.12)
Certificate and above	77	63	0.978 (0.60, 1.60)	
Maternal occupation status				
Housewife	95	86	1	1
Government Employee	61	57	1.010 (1.08, 3.31)	0.510 (0.15, 1.79)
Merchants	47	42	1.020 (0.49, 2.13)	0.760 (0.18, 3.26)
Others	8	7	1.470 (0.75, 2.89)	0.840 (0.19, 3.66)
Occupation status of the father				
Government employee	74	71	1	1
Merchant	62	63	1.698 (0.80, 3.81)	1.900 (0.47, 7.72)
Self-employee	52	45	1.798 (0.79, 3.15)	1.970 (0.48, 0.14)
Others	23	13	1.531 (1.14, 4.07)	1.820 (0.34, 9.78)
Birth interval of the mothers				
<24 Months	58	74	**1**	**1**
>24 Months	79	42	**2.400 (1.44, 3.99)**	**2.850(1.45, 4.25)**
Number of children under 5 years				
One	165	142	1	1
Two	46	50	0.792 (0.50, 1.25)	0.970 (0.48, 1.14)
Family size				
2–3 members	61	52	1	1
4–5 members	95	80	0.781 (0.46, 1.31)	1.230 (0.69, 2.18)
6 and above members	55	60	0.772 (0.48, 1.24)	1.560 (1.05, 2.31)
Food security				
Food security	116	133	**1**	**1**
Food insecure	95	59	**1.846 (1.23, 2.78)**	**2.230 (1.63, 3.10)**

Note: The “bold” value indicates there is a significant association with the outcome variables.

## Discussion

The aim of this study was to assess the dietary diversity of children aged 6–23 months and identify associated factors that influence dietary diversity. Adequate dietary diversity for children aged 6–23 months is crucial for maintaining optimal health and promoting normal growth and development. Various factors influence the DDs of children, and this causes health problems and affects the growth and development of the child. According to the essential nutrition action (ENA), existing studies recommended that adequate dietary diversity during this age group was very crucial for normal growth and development (36).

In this finding, 192 (47.6%) children aged 6–23 had adequate dietary diversity, which is higher than that observed in a study conducted in Northwest Ethiopia (18.2%), the Ethiopian Demographic and Health Survey 2016 (12.09%), Southern Ethiopia (10.6%), Chelia District, Ethiopia (17.32%), Gedeo Zone, Ethiopia (29.9%), East Africa (10.47%), and sub-Saharan Africa (SSA) (25.1%) (2, 6, 37–40). Several factors may contribute to this variation in findings.

The potential cause might be variations in sample size, and the study design can influence the results; for example, larger sample sizes or different methodologies might yield different outcomes.

The approach to measuring dietary diversity, including the accuracy of self-reported data and recall periods, agricultural practices, such as local crop availability and farming techniques, environmental factors, including regional climate and economic conditions, cultural dietary practices, and socio-economic conditions, plays a role in determining dietary patterns. Understanding these factors can provide context for the observed differences and underscore the importance of considering local conditions when interpreting dietary diversity in the 24 h during the survey.

Similarly, this discrepancy may be attributed to differences in measurement of DDs, the category of food group, and the study setting, i.e., some studies use seven food groups, and if four food groups were consumed from the seven, they classified as adequate dietary diversity; however in our study, we used eight food groups, and we classified them as adequate dietary diversity if they consumed five from eight food groups.

The results of this finding are almost consistent with the those of a study conducted in Addis Ababa, Ethiopia (34), Southern Ethiopia (41), Bale Zone, Southern Ethiopia (42), Haramaya, Ethiopia (43), Northwest Ethiopia (44), and Bangladesh (34). The similarity of the findings across the studies may be attributed to the fact that the majority of these studies were conducted in Ethiopia. This shared geographical and cultural context likely results in similar socio-demographic, socio-economic, and seasonal variations, which can influence dietary diversity in comparable ways.

The common socio-economic conditions, agricultural practices, and seasonal availability of food in Ethiopia contribute to the observed similarities in dietary diversity rates.

On the other hand, the finding of this study is lower as compared with another study that is done in Indonesia (Indonesia Demographic and Health Survey) (53·1%) in 2007, (51·7%) in 2012, and (53·7%) in 2017 (45). This might be explained by the difference in the study period, which can result in food security status change, as well as socio-demographic, socio-cultural, and geographical variations.

The study results might vary due to differences in self-reported measurement and recalling food given in the 24 h before the survey. In addition, information accessible area, time of the study, and related socio-economic characteristics could also affect the estimated minimum dietary diversity score.

In our study, the mother’s educational status was one significant variable for the MDDs of the child. Mothers with a secondary education and above were two times more likely to have good dietary diversity for their children as compared to those with less than secondary education.

Similarly, the study in Chelia District, Ethiopia (6), the study in Gorche District, Southern Ethiopia (46), the study in Indonesia (45), the study in East Africa (40), and the study in Wolaita Sodo, Southern Ethiopia, showed that illiterate mothers were less likely to feed their children to fulfill the minimum requirement of dietary diversity of food for their children. This might be a lack of understanding and knowledge of the importance of MDDs for the normal health and both growth and development of the child.

In this finding, birth interval is one pertinent and significant predictor of normal MDDs of a child aged 6–23 months. Mothers with birth intervals greater than 24 months were three times more likely to practice good dietary diversity than those mothers with birth intervals less than 24 months{{Mekonnen et al. (47) #8}Sema et al. (20) #7{Anane et al. (48) #4}}.

In general, the optimal breastfeeding is 2 years, and if there is a birth of fewer than 2 years, then the first baby does not get adequate parental care including MDDs; in this age group, the parent will give their attention to the new baby. In addition, the mothers have no time to give adequate care for their two little children (49), and that may challenge their economy as well as they may have fewer chances of meeting nutrient requirements for the child for feeding the child based on the recommendations (20, 50). Mothers who had food insecurity were 2.23 times more likely to have adequate dietary diversity for their children as compared to their counterparts. Similarly, in a study in Mali, Gorche District, Southern Ethiopia, EDHS, 2016, and Debub Bench Zone, women from extremely food-insecure households were less likely to practice good MDDs for their children (46, 51–54). When women have food security, they become more concerned with adequate dietary diversity (MDDs) and immediately can put them into practice. This is supported by a study conducted in Boston; food insecurity may worsen diet quality and diversity (45, 54, 55).

## Conclusion and recommendation

The study revealed that nearly half of the participants fail to meet the minimum dietary diversity (MDD) standards recommended by the WHO-UNICEF Technical Expert Advisory Group. This underscores the urgent need for intervention to address the prevalent issue of inadequate MDDs among children. Factors such as maternal educational level, short birth intervals, and household food insecurity emerged as significant predictors of insufficient MDDs in this research. The findings can inform public health policy and practice by guiding targeted local interventions, shaping national regulations, adjusting funding priorities, and updating evidence-based guidelines, thus enhancing both immediate and long-term health outcomes.

We recommended that stakeholders, including the Ministry of Health, regional health offices, and agricultural sectors, prioritize the enhancement of child nutrition through targeted food-based approaches. Developing and implementing comprehensive intervention programs to improve children’s minimum dietary diversity (MDD) should be a central focus. Health professionals should strengthen nutritional education to promote adequate dietary diversity (MDD) practices. This effort should not rest solely on government bodies but must involve collaboration across all relevant sectors, including the Ministry of Education, healthcare providers, community organizations, and every household. Such a multi-faceted approach is essential to effectively improving nutritional outcomes for children aged 6–23 months. In addition, health personnel should be actively engaged in family planning and educating families on the importance of MDD for child health.

### Strengths and limitations

The strength of the study was the use of standardized and validated measurement tools, which enhanced the accuracy and reliability of the DD data. In addition, the provision of training to the data collectors ensures that data collection is consistent and high-quality. Despite having a representative sample, the cross-sectional design limits the study’s ability to establish causality or track changes over time. Furthermore, recall bias may affect the accuracy of reported dietary practices and predictors as participants may not always accurately remember or report their dietary behaviors.

## Data Availability

The original contributions presented in the study are included in the article/supplementary material, further inquiries can be directed to the corresponding author.

## Glossary

CDC: Central for Disease Control
CI: confidence interval
DD: dietary diversity
DDS: dietary diversity score
DHS: Demographic and Health Survey
EDHS: Ethiopia Demographic Health Survey
EPHIA: Ethiopia Population-based HIV Impact Assessment
FANTA: Food and Nutrition Technical Assistance Project
FANTA: Food and Nutrition Technical Assistance
HDA: Health Development Army
HEWs: health extension workers
HFIAS: Household Food Insecurity Assessment Scale
HH: household
IRB: Institutional Review Board
IYCF: infant and young child feeding
MCH: maternal and child health
MDDS: minimum dietary diversity score
MOH: Ministry of Health
NNP: National Nutrition Program
PCA: principal component analysis
PNC: postnatal care
